# Supplementary material for: The effects of genital myiasis on the diversity of the vaginal microbiota in female Bactrian camels
Source: BMC Vet Res. 2022 Mar 5;18:87. doi: 10.1186/s12917-022-03189-5 (PMC8897907; doi:10.1186/s12917-022-03189-5)
Supplement: Supplementary file 5 — Additional file 5. [file 12917_2022_3189_MOESM5_ESM.zip › MPL201709200_16s_yy/Treat1/css/js_css/jsPDF-master.zip!/jsPDF-master/docs/FontObject.html]

FontObject - Documentation


## Home

### Classes

- FontObject
- jsPDF
- PubSub

### Global

- addFont
- addMetadata
- addPage
- CapJoinStyles
- circle
- CssColors
- ellipse
- getFontList
- html2pdf
- http
- lines
- lstext
- output
- rect
- requirejs
- reset
- roundedRect
- save
- setDisplayMode
- setDrawColor
- setFillColor
- setFont
- setFontSize
- setFontStyle
- setLineCap
- setLineJoin
- setLineWidth
- setPage
- setProperties
- setTextColor
- text
- toUrl
- triangle
- triggerEvent

# FontObject

## FontObject

#### new FontObject()

Source:
:   - jspdf.js, line 419

##### Properties:

| Name | Type | Description |
| --- | --- | --- |
| `id` | String | PDF-document-instance-specific label assinged to the font. |
| `PostScriptName` | String | PDF specification full name for the font |
| `encoding` | Object | Encoding\_name-to-Font\_metrics\_object mapping. |

FontObject describes a particular font as member of an instnace of jsPDF

It's a collection of properties like 'id' (to be used in PDF stream),
'fontName' (font's family name), 'fontStyle' (font's style variant label)

  

Documentation generated by JSDoc 3.4.2 on Sat Oct 08 2016 21:42:26 GMT+0100 (BST) using the docdash theme.
